# Mirtazapine added to selective serotonin reuptake inhibitors for treatment-resistant depression in primary care (MIR trial): study protocol for a randomised controlled trial

**DOI:** 10.1186/s13063-016-1199-2

**Published:** 2016-02-03

**Authors:** Debbie Tallon, Nicola Wiles, John Campbell, Carolyn Chew-Graham, Chris Dickens, Una Macleod, Tim J. Peters, Glyn Lewis, Ian M. Anderson, Simon Gilbody, William Hollingworth, Simon Davies, David Kessler

**Affiliations:** 1 School of Social and Community Medicine, University of Bristol, Oakfield House, Oakfield Grove, Bristol, BS8 2BN UK; 2 University of Exeter Medical School, St Luke’s Campus, Smeall Building, Magdalen Road, Exeter, EX1 2LU UK; 3 Research Institute for Primary Care and Health Sciences, Keele University, Keele, Staffordshire ST5 5BG UK; 4 University of Exeter Medical School, Room 1.04, College House, St Luke’s Campus, Heavitree Road, Exeter, EX1 2LU UK; 5 Hull York Medical School, University of Hull, Kingston upon Hull, HU6 7RX UK; 6 School of Clinical Sciences, 69 St Michael’s Hill, Bristol, BS2 8DZ UK; 7 University College London, Maple House, 149 Tottenham Court Rd, London, W1T 7NF UK; 8 Neuroscience and Psychiatry Unit, The University of Manchester, Room G809, Stopford Building, Oxford Road, Manchester, M13 9PT UK; 9 Mental Health Research Group, Department of Health Sciences and Hull York Medical School, Alcuin College C Block, University of York, YO10 5DD Heslington, UK; 10 School of Social and Community Medicine, University of Bristol, Canynge Hall, 39 Whatley Road, Bristol, BS8 2PS UK; 11 Centre for Addiction and Mental Health, Room 6318, 80 Workman Way, Toronto, ON Canada

**Keywords:** Depression, Treatment-resistant, Antidepressants, Mirtazapine, Selective serotonin reuptake inhibitors

## Abstract

**Background:**

People with depression are usually managed in primary care and antidepressants are often the first-line treatment, but only one third of patients respond fully to a single antidepressant. This paper describes the protocol for a randomised controlled trial (MIR) to investigate the extent to which the addition of the antidepressant mirtazapine is effective in reducing the symptoms of depression compared with placebo in patients who are still depressed after they have been treated with a selective serotonin reuptake inhibitor (SSRI) or serotonin and noradrenaline reuptake inhibitor (SNRI) for at least 6 weeks in primary care.

**Methods/Design:**

MIR is a two-parallel group, multi-centre, pragmatic, placebo controlled, randomised trial with allocation at the level of the individual. Eligible participants are those who: are aged 18 years or older; are currently taking an SSRI/SNRI antidepressant (for at least 6 weeks at an adequate dose); score ≥14 on the Beck Depression Inventory (BDI-II); have adhered to their medication; and meet ICD-10 criteria for depression (assessed using the Clinical Interview Schedule-Revised version).

Participants who give written, informed consent, will be randomised to receive either oral mirtazapine or matched placebo, starting at 15 mg daily for 2 weeks and increasing to 30 mg daily thereafter, for up to 12 months (to be taken in addition to their usual antidepressant). Participants, their GPs, and the research team will all be blind to the allocation. The primary outcome will be depression symptoms at 12 weeks post randomisation, measured as a continuous variable using the BDI-II.

Secondary outcomes (measured at 12, 24 and 52 weeks) include: response (reduction in depressive symptoms (BDI-II score) of at least 50 % compared to baseline); remission of depression symptoms (BDI-II <10); change in anxiety symptoms; adverse effects; quality of life; adherence to antidepressant medication; health and social care use, time off work and cost-effectiveness. All outcomes will be analysed on an intention-to-treat basis.

A qualitative study will explore patients’ views and experiences of either taking two antidepressants, or an antidepressant and a placebo; and GPs’ views on prescribing a second antidepressant in this patient group.

**Discussion:**

The MIR trial will provide evidence on the clinical and cost-effectiveness of mirtazapine as an adjunct to SSRI/SNRI antidepressants for patients in primary care who have not responded to monotherapy.

**Trial registration:**

EudraCT Number: 2012-000090-23 (Registered January 2012); ISRCTN06653773 (Registered September 2012)

## Background

Depression is ranked amongst the top five contributors to the global burden of disease, and by 2030 is predicted to be the leading cause of disability in high income countries [1]. People with depression are usually managed in primary care and antidepressants are usually the first-line treatment. The number of prescriptions for antidepressants has risen dramatically in recent years; increasing by 7.2 % (3.8 million items) between 2013 and 2014. Indeed, antidepressants have shown a greater increase in the volume of prescribing in 2014 than drugs for any other therapeutic area, with over 57 million prescriptions being issued in England in 2014, at a cost of £265 million [2].

However, the STAR*D study (Sequenced Treatment Alternatives to Relieve Depression) found that half of those treated did not experience at least a 50 % reduction in depressive symptoms following 12–14 weeks of treatment with a single antidepressant [3]. The reasons for this non-response are complex but include what can be termed treatment-resistant depression (TRD), where an adequate dose and duration of treatment has been taken.

When first-line antidepressant treatments do not work, general practitioners (GPs) can be unsure what to offer next. The National Institute for Health and Care Excellence (NICE) now advises GPs to reconsider the treatment option if there has been no response after 4—6 weeks of antidepressant medication [4]. However, there is currently limited evidence to guide management.

### Existing evidence on the pharmacological management of treatment-resistant depression

The current NICE guideline [4] describes the following pharmacological strategies for sequencing treatments after inadequate response to initial treatments: switching antidepressants; augmenting medication by adding a drug which is not an antidepressant; and combining antidepressants. The guidelines comment in general on the lack of evidence and particularly that ‘the evidence for the relative advantage of switching either within or between classes is weak’. Connolly et al. comment that switching antidepressants after inadequate response is not ‘unequivocally supported by the data, although switching from a selective serotonin receptor inhibitor (SSRI) to venlafaxine or mirtazapine may … offer greater benefits’ [5]. Similarly, there is very little evidence on combining two antidepressants.

The evidence for the effectiveness of augmentation with a non-antidepressant is likewise of variable quality. There is some evidence for augmentation with lithium or thyroid hormone, but mainly in combination with tricyclic antidepressants, which are prescribed much less often today. The use of the atypical antipsychotic drugs to augment the newer antidepressants is better supported by research [6, 7], with quetiapine and aripiprazole the most promising [8]. However, this combination has not to date been adopted with any enthusiasm in UK primary care. This may be because of a lack of experience in prescribing them for this indication since they are usually initiated in secondary care, as well as concerns about their adverse effects [9] (e.g. sedation, metabolic syndrome and central obesity, extrapyramidal side effects.) Indeed, the current NICE guidance is that antidepressants should not be combined or augmented without the advice of a consultant psychiatrist [4].

It is possible that GPs would consider adding a second antidepressant, rather than an atypical antipsychotic drug or lithium, as part of the management of TRD. They are more familiar with these drugs and their starting routines and there is less concern about their adverse effects, and less need for monitoring. In general, stepwise combination of drug treatments is a standard part of the management of chronic diseases in primary care such as asthma and hypertension and has led to improved clinical outcomes. GPs are comfortable with this model of care and would probably readily adopt this strategy if it were found to be effective. We think there may be an opportunity to substantially improve the treatment of depression in primary care by using antidepressants in combination. However, one of the reasons that this strategy has not been adopted is the lack of convincing evidence for its effectiveness, especially in the primary care setting.

There is a pharmacological rationale for adding a second antidepressant to SSRIs or serotonin and noradrenaline reuptake inhibitors (SNRIs) with a different and complementary mode of action. Mirtazapine, an alpha_2_-adrenoreceptor antagonist, increases central noradrenergic and serotonergic neurotransmission by inhibiting negative feedback from synaptic noradrenaline (NA) acting on presyaptic alpha_2_-autoreceptors on NAergic neurones and alpha_2_-heteroreceptors on 5-hydroxytryptaminergic (5-HTergic) neurones. Its mechanism of action is, therefore, different to that of both SSRIs and SNRIs which inhibit synaptic neurotransmitter reuptake after release. Thus, treatment with mirtazapine in combination with either an SSRI or SNRI may produce a sustained increase in both 5-HT and NA synaptic availability in terminal fields. A further property of mirtazapine not shared by SSRIs and SNRIs is an affinity for the 5HT-2C receptor where it acts as an inverse agonist. This mechanism has been linked to specific therapeutic effects. Overall, there is the potential for a synergistic action that could enhance clinical response compared to those patients receiving only monotherapy. Mirtazapine is now off patent and relatively inexpensive.

Because of its different mechanism of action there is an argument that switching to mirtazapine alone after SSRI treatment failure might be an effective strategy, rather than subjecting patients to the potential adverse effect burden of a second medication. The STAR*D study compared mirtazapine to nortriptyline in a group of patients who had not responded to two consecutive antidepressant monotherapy regimes. The rates of remission were low for both drugs [10], suggesting that switching to mirtazapine monotherapy is not the most useful strategy.

In spite of the potential benefit of combining mirtazapine with an SSRI there is relatively little trial evidence supporting this strategy. Carpenter et al. compared the addition of mirtazapine to an SSRI with placebo in a group of 26 patients who had not responded to at least 4 weeks of monotherapy. Although the sample size was very small, the results in terms of effectiveness and tolerability are encouraging [11] but more definitive evidence is required before widespread adoption. In patients who have not failed previous treatment, Blier et al. reported that mirtazapine in combination with an SSRI gave a greater improvement than monotherapy [12], and that it was well-tolerated with either an SSRI or an SNRI (venlafaxine) [13], with both combinations providing significantly higher remission rates than an SSRI alone. In contrast a larger study found no benefit from combining antidepressants, including mirtazapine and venlafaxine, over SSRI monotherapy with escitalopram [14], though combined treatment had a higher side-effect burden.

Mirtazapine treatment is, however, associated with more weight gain than SSRIs [15] and, therefore, as well as assessing the efficacy of its combination with SSRIs it is important to determine its adverse effect burden, especially in long-term treatment.

### Defining treatment-resistant depression (TRD)

Many definitions of treatment resistance have been proposed. These definitions cover a broad spectrum ranging from failure to respond to at least 4 weeks of antidepressant medication given at an adequate dose [16] to more stringent criteria based on non-response to multiple courses of treatment [5].

We have used a more inclusive definition of TRD; that is patients who still meet the *International Classification of Diseases* (ICD-10) criteria for depression after taking an SSRI or SNRI antidepressant at an adequate dose (based on the *British National Formulary* (BNF) [17] and advice from psychopharmacology experts), for a minimum of 6 weeks. This definition is directly relevant to UK primary care, given the uncertainty about what course of action to recommend to this group of patients.

Although this 6-week criterion seems a relatively short period to define treatment resistance, many of the patients who satisfy this criterion of ‘non-response’ are suffering from moderate to severe chronic depression. The baseline measures for a recent study of the effectiveness of cognitive behavioural therapy (CBT) for treatment-resistant depression in primary care, the CoBalT study [18], found that 59 % of those recruited had been depressed for more than 2 years; that 70 % had been prescribed their current antidepressant for more than 12 months; and that 28 % satisfied the ICD-10 criteria [19] for severe depression. These data on chronicity and severity illustrate the extent of the unmet need in this population [20].

At present, there is no good evidence that switching antidepressants improves outcomes. For this reason the latest NICE guideline update [21] emphasises considering alternative strategies, such as augmentation, after a single failure of antidepressants.

It is, therefore, important to undertake a study to investigate the effectiveness of the addition of mirtazapine to SSRIs or SNRIs in primary care. In the UK, most depression is diagnosed and treated in primary care, and this is where most antidepressants are prescribed, and most treatment resistance encountered. The rise in antidepressant prescribing has continued at a steady rate in the UK despite the introduction of the government’s initiative to Improve Access to Psychological Therapies (IAPT). Failure to adequately respond to treatment is a substantial problem and there is a need to develop the evidence base for the rational prescribing of antidepressants in primary care. An effective intervention has the potential to have a substantial impact on the health and economic burden associated with this patient group.

### Objective

The trial will investigate whether combining mirtazapine with SNRI or SSRI antidepressants results in better patient outcomes and more efficient NHS care than SNRI or SSRI therapy alone in TRD. All patients entering the trial will be recruited from primary care and will have treatment-resistant depression, defined as meeting ICD-10 [19] criteria for depression after at least 6 weeks treatment with either an SSRI or SNRI antidepressant at an adequate dose.

Our specific aims are to:

1. Determine the effectiveness of the addition of the antidepressant mirtazapine to an SSRI or SNRI in reducing depressive symptoms and improving quality of life at 12 weeks, 24 weeks and 12 months (compared to the addition of a placebo)
2. Determine the cost-effectiveness of this intervention over 12 months
3. Qualitatively (a) explore patients’ views and experiences of taking either two antidepressant medications or an antidepressant and a placebo; (b) identify patients’ reasons for completing or not completing the study, including withdrawal from study medication
4. Qualitatively explore GPs’ views on prescribing combined antidepressant therapy in this patient group

## Methods/Design

### Study design

The MIR study is a two-parallel group multi-centre pragmatic placebo controlled randomised trial with allocation at the level of the individual. Patients will be recruited from general practices in England, in the areas surrounding our four recruiting centres: Bristol, Exeter, Hull and Keele. The primary outcome will be at 12 weeks. The Beck Depression Inventory (BDI-II) was selected as the primary outcome, rather than a clinician-rated instrument, to avoid the potential for observer bias inherent in using clinician-rated instruments. The double-blinded randomised allocation will be maintained for a period of 12 months, although participants can be unblinded at their request or the request of their GP after the primary outcome at 12 weeks, and outcomes will also be measured at 24 weeks and 12 months. These include cost-effectiveness which will be assessed at 12 months. A nested qualitative study will explore patients’ and GPs’ views of the use of an additional antidepressant.

Ethical approval was obtained from South East Wales Research Ethics Committee Panel C (ref: 12/WA/0353); Bristol Clinical Commissioning Group (CCG), and other relevant CCGs provided research governance assurance. Clinical trial authorisation was provided by the Medicines and Healthcare products Regulatory Agency. The trial sponsor is the University of Bristol. The trial has been registered: EudraCT Number: 2012-000090-23 (January 2012); ISRCTN06653773 (September 2012).

### Inclusion and exclusion criteria

#### Inclusion criteria

Primary care patients who: are aged 18 years or older; have depression treated for at least 6 weeks with any one of the following SSRI or SNRI antidepressants at recommended BNF doses: fluoxetine, sertraline, citalopram, escitalopram, fluvoxamine, paroxetine, duloxetine, or venlafaxine. The minimum SSRI and SNRI dose criteria were based on the BNF and on advice from psychopharmacology experts, and have been used in earlier trials of TRD [22]. Table 1 lists the adequate dose criteria used in the MIR study. To be eligible, participants must also have adhered to their medication. While adherence to medication is difficult to measure, we will operationalise our definition of treatment resistance by using the Morisky 4-item self-report measure of compliance [23] as adapted for the CoBalT trial [22]. The Morisky measure has previously been validated against electronic monitoring bottles, with a score of zero (range: 0–4) indicating at least 80 % compliance [24]. Given the relatively long half-life of antidepressant medication, individuals who have forgotten to take one or two tablets will not be excluded. Participants also need to score at least 14 on the BDI-II [25], and have an ICD-10 diagnosis of depression (assessed using the Clinical Interview Schedule-Revised version (CIS-R) [26].

**Table 1:** Adequate doses for selective serotonin receptor inhibitor (SSRI) and serotonin and noradrenaline receptor inhibitor (SNRI) antidepressants – table showing the list of adequate doses of SSRI/SNRI antidepressants used in the MIR study

Name	Trade name	Type	BNF^a^ Code	Minimum adequate daily dose (mg)
Citalopram	Cipramil	SSRI	4.3.3	20
Duloxetine	Cymbalta/ Yentreve	SNRI	4.3.4	60
Escitalopram	Cipralex	SSRI	4.3.3	10
Fluoxetine	Prozac	SSRI	4.3.3	20
Fluvoxamine	Faverin	SSRI	4.3.3	100
Paroxetine	Seroxat	SSRI	4.3.3	20
Sertraline	Lustral	SSRI	4.3.3	100
Venlafazine	Effexor	SNRI	4.3.4	75

^a^Source: *British National Formulary* (BNF) No.55 (March 2008) for BNF code and dosage. These minimum doses were chosen to reflect prescribing guidance in UK primary care, and with expert advice from psychopharmacologists

#### Exclusion criteria

GPs will be asked to exclude patients who are: currently taking combined or augmented antidepressant treatment; are having their medication managed by a psychiatrist; have dementia (formal diagnosis), bipolar disorder, psychosis or alcohol/substance abuse/dependence; are pregnant, planning pregnancy, or breast-feeding; are unable to complete the study questionnaires; have had a previous adverse reaction to mirtazapine; have current treatment with a monoamine oxidase (MAO) inhibitor including moclobemide; or have other medical contraindications to mirtazapine.

### Recruitment of participants

The trial will recruit participants from primary care who have depression that has not responded to at least 6 weeks treatment with SSRI or SNRI antidepressants, prescribed at an adequate therapeutic dose. We plan to recruit 470 patients over 18 months from 96 practices linked with our four recruitment centres, using two methods of recruitment: record search and in-consultation recruitment.

### Method 1: record search

GP practices will search their computerised records for potentially eligible patients; those who have been prescribed SSRI or SNRI antidepressants for at least 6 weeks at an adequate dose (Table 1), as recommended in the BNF [17]. These patients will then be mailed an invitation by their GP to participate, asking for their permission to be contacted by the research team. Patients who have not responded after 2 weeks will be sent one reminder letter by the practice. GPs will be asked to provide anonymised data on the age and gender of those patients who were mailed an invitation to participate (including those who did not respond) in order to assess the generalisability of the findings.

### Method 2: in consultation

GPs can also identify potentially suitable patients in consultation. They will introduce the trial and ask the patient for their written consent to be contacted by the research team.

### Postal screening

Patients who have agreed to be contacted by either method of recruitment will be sent a postal screening questionnaire. This will contain a BDI-II, a measure of adherence to antidepressant medication (Morisky), a question about duration of medicine use, demographic questions, and a list of the main exclusion criteria. The GPs will be asked to review all those who appear eligible on the postal screening, and to confirm that they are suitable to be prescribed mirtazapine. Once GP approval is received, patients will be invited to a baseline assessment with a researcher, either in their own home or at their general practice.

### Baseline assessment

The researcher will explain the study in detail and obtain written informed consent for the baseline assessment. If the potential participant is agreeable they will complete the following questionnaires: BDI-II; Morisky (adherence to medication); Patient Health Questionnaire (PHQ9) [27] a brief measure of depression; CIS-R – an in-depth psychiatric questionnaire which gives an ICD-10 diagnosis. Participants will be asked for details of their prescribed medication, prior use of antidepressants and whether they are currently in receipt of psychological therapy. In addition, socio-demographic details will be recorded (age, gender, ethnicity, marital status), together with information on a number of socio-economic markers (employment status, housing situation).

Potential participants who score 14 or more on the BDI-II, and who have an ICD-10 primary diagnosis of depression using the CIS-R, will be told they are potentially eligible to enter the trial (pending confirmation by the principal investigator (PI)) and will be asked to provide further written consent for trial participation, and to indicate whether they would be willing to be contacted about future research projects.

These potentially eligible participants will also be asked to complete some further questionnaires including: the General Anxiety Disorder questionnaire (GAD-7) [28]; EQ-5D-5 L [29], a brief measure of health-related quality of life; SF-12, a brief measure of mental and physical functioning; ASEC measure of antidepressant side effects [30]. They will be asked further questions about their history of depression and whether they have ever been referred to a psychiatrist; and about the strength of their preference for active treatment over placebo (as this may potentially affect medication adherence and outcomes). Additional information will be collected on life events, financial stress, social support and alcohol use [31].

Once the baseline assessment is complete, the local research clinician (PI) will review the baseline information and confirm whether the patient is eligible for the MIR trial.

### Randomisation procedure and code break (unblinding)

Following the baseline assessment, eligible and consenting participants will be randomised using the automated randomisation service provided by Bristol Randomised Trials Collaboration. Randomisation will be by means of a computer-generated code to ensure concealment of allocation.

Participants are randomly assigned to one of two treatments: (1) one × 15 mg encapsulated mirtazapine daily for 2 weeks followed by two × 15 mg encapsulated mirtazapine for up to 50 weeks; or (2) identical placebo.

Randomisation will be stratified by centre (*n* = 4) to ensure a balance in terms of local differences. Minimisation will be used to ensure balance in baseline BDI-II score (using approximate tertiles derived from the CoBalT baseline scores; <26; 26–34, ≥35), gender and whether the patient is currently receiving a psychological therapy; ensuring a balance in these important prognostic indicators. We will use minimisation with a probability weighting of 0.8 in order to reduce predictability [32].

Patient packs containing the trial medication will be sent to the participant’s GP (or home in exceptional circumstances) at regular 6–8 week intervals.

Participants are free to withdraw from the medication at any time. Patients, clinicians, outcome assessors, data analysts and the rest of the research team will be blinded to allocation. All patients continue with their GP care and usual antidepressant as agreed by their GP. Clinicians will not be restricted in their use of psychological services.

Unblinding will be available via the trial pharmacy at all times in case of a medical emergency (‘emergency unblinding’). After the 12-week primary outcome has been completed, the code can also be broken at the request of the participant or their GP (‘routine unblinding’). Those who have not requested emergency or routine unblinding, will be unblinded at the end of the follow-up period, or on withdrawal from the study. The trial team will not provide further supplies of the trial medication once participants have been unblinded.

### Follow-up schedule

At 2 weeks post - baseline, researchers will contact participants briefly by telephone to check they have received and started their trial medication.

**Fig. 1:**
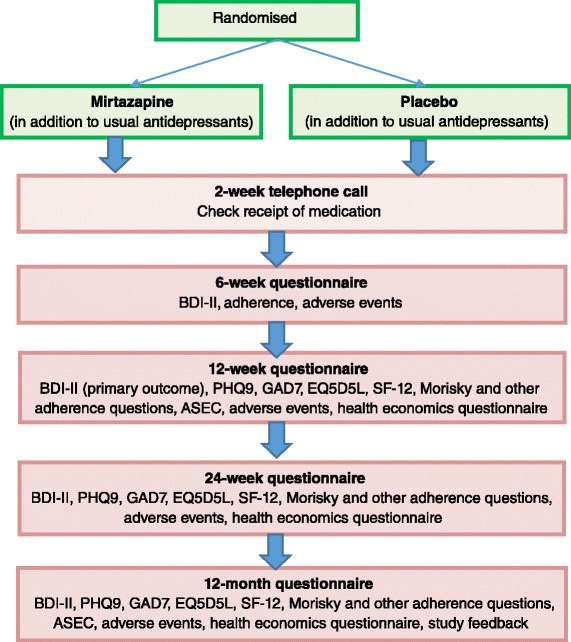
Summary of the follow-up schedule – flow-chart illustrating the follow-up schedule for the MIR study

At 6, 12, 24 and 52 weeks, participants will be asked to complete self-report outcome questionnaires. The follow-up schedule is summarised in the flow-chart (Fig. 1) and table (Table 2). Follow-up questionnaires can be completed face-to-face with the researcher, by phone, or by post. If a postal questionnaire is not returned, a reminder will be sent.

**Table 2:** Full schedule of questionnaires – table showing the questionnaires scheduled for the MIR study

Questionnaire	Postal screening	Baseline	2 weeks call	6 weeks	12 weeks	24 weeks	12 months
Consent form		Y					
BDI-II	Y	Y		Y	Y	Y	Y
Biographic and demographic data including psychiatric history, life events, social support, alcohol use	Y	Y					
Views on treatment		Y					
Assessment of blinding					Y		
Medication	Y	Y		Y	Y	Y	Y
Morisky (adapted)	Y	Y		Y	Y	Y	Y
CIS-R		Y					
PHQ9		Y			Y	Y	Y
GAD-7		Y			Y	Y	Y
EQ-5D-5L		Y			Y	Y	Y
SF-12		Y			Y	Y	Y
Economics					Y	Y	Y
Health events (SAEs)				Y	Y	Y	Y
ASEC		Y			Y		Y
2-week check			Y				
Blinding questions					Y		
Exit questionnaire							Y

*ASEC* Antidepressant Side Effect Checklist, *BDI-II* Beck Depression Inventory II, *CIS-R* Clinical Interview Schedule-Revised version, *EQ-5D-5L* EuroQol 5 dimensions 5 levels quality of life questionnaire, *GAD-7* Generalised Anxiety Disorder-7 questionnaire, *PHQ9* Patient Health Questionnaire, *SAE* Serious Adverse Event, *SF-12* social and physical functioning questionnaire short form 12

Throughout the follow-up process participants are asked about possible adverse effects and advised to consult their GP about these if appropriate. Participants will be sent a £5 gift voucher with each of the 12-week, 24-week, and 12-month questionnaires, to thank them for their participation.

At the end of the 12-month follow-up period, (or on withdrawal from the study), participants will be advised to return to their GP to discuss their continued care.

### Withdrawal of trial participants

Participants can withdraw from the trial at any time for any reason, without their medical care being affected. Where possible, data already collected will continue to be used in the trial and patients who withdraw from the trial will be asked if they are still willing to provide follow-up data. If a patient withdraws, the reason for, and type of, withdrawal will be documented in the Case Report Form (CRF).

PIs have the right to withdraw patients from the trial drug in the event of inter-current illness, Adverse Events (AEs), Serious Adverse Events (SAEs), Suspected Unexpected Serious Adverse Reactions (SUSARs), protocol violations, administrative reasons or other reasons; this will be documented in the CRF.

Although there is no evidence that the medication is teratogenic, if a patient discovers that she is pregnant during the trial she will be instructed to stop her trial medications immediately, though she will be able to continue to participate in completion of the trial outcome measures if she wishes. A longer monitoring period will be put in place to establish the safe delivery of a healthy infant, at which point follow-up will stop.

### Outcome measures

Primary outcomes:

Continuous BDI-II score at 12 weeks, adjusting for baseline.

Secondary outcomes:

1. Treatment response, measured as an improvement of at least 50 % in BDI-II score at 12 weeks compared with baseline
2. The rate of remission of symptoms, defined as a score on the BDI-II of less than 10 at 12 weeks
3. Change in anxiety symptoms (measured with GAD-7) at 12 weeks
4. All of the above outcomes at 24 weeks and 12 months
5. Antidepressant use and adherence (using Morisky and additional questions)
6. Quality of life (using the EQ-5D-5L) and social and physical functioning (SF-12), at 12 weeks, 24 weeks and 12 months
7. Adverse Events including: any new symptoms or worsening of existing symptoms, consultations for a documented deterioration in illness and Serious Adverse Events (self-reported, or from primary care notes review); adverse effects (using the Antidepressant Side Effect Checklist (ASEC) [30] at 12 weeks and 12 months)
8. Cost-effectiveness from the perspectives of the NHS, patients, and society (using self-report questionnaires at 12 and 24 weeks, and at 12 months; and primary care practice data on consultations, services and prescriptions over the 12-month trial period)

### Trial medication

The active trial drug will be mirtazapine: 1 × 15 mg oral capsule per day for 2 weeks followed by 2 × 15 mg oral capsules per day for up to 12 months. The mirtazapine will be encapsulated and the placebo will be an identical capsule filled with an inert excipient. The placebo capsule will exactly match the encapsulated mirtazapine in dimensions and appearance, so that allocation concealment and blinding of the trial is maintained.

### Packaging, labelling and dispensing

The labelling of medication packs will be Medicines and Healthcare products Regulatory Authority (MHRA) approved and conform to Annexe 13 of Good Manufacturing Practice (GMP) standards and Article 13.3 of Directive 2001/20/EC [33]. Each medication pack will have a medicine identification number, randomly generated to ensure mirtazapine and placebo medicine packs are indistinguishable and thus maintain allocation concealment. This random number will be generated by the Bristol Randomised Trials Collaboration and provided to the manufacturer who will use it to form the identifier.

Sharp Clinical Services will provide Qualified Person (QP) services and distribution and project management. They will ship labelled and numbered packages to the central trial pharmacy (University Hospitals Bristol (UHB) Clinical Trials Pharmacy), where the trial medication will be stored under controlled conditions. Storage will be secure, and there will be a delegation log for access, for which the trial pharmacy will take responsibility. The trial pharmacy will dispense individual patient packs and oversee the packaging and posting of those packs. Patient packs containing no more than an 8-week supply of the trial medication will be posted by recorded delivery. All deliveries will be logged to ensure drug accountability. The trial medication will be shipped and stored in conditions in line with manufacturer’s stability data.

### Concomitant medication

Pharmacodynamic interactions:

Mirtazapine should not be administered concomitantly with MAO inhibitors or within 2 weeks after discontinuation of MAO inhibitor therapy. Likewise about 2 weeks should pass before patients treated with mirtazapine should be treated with MAO inhibitors. Participants in this study will not be treated with MAO inhibitors and GPs will be advised to wait at least 2 weeks after stopping the trial medication before starting an MAO inhibitor.

Co-administration with other serotonergic active substances (L-tryptophan, triptans, tramadol, linezolid, lithium and St. John’s Wort – *Hypericum perforatum* – preparations) may lead to an incidence of serotonin-associated effects and participants will be advised to use these medications with caution. Mirtazapine may increase the sedating properties of benzodiazepines and other sedatives (notably most antipsychotic drugs, antihistamine H1 antagonists, opioids). Caution should be exercised when these medicinal products are prescribed together with mirtazapine.

Mirtazapine may increase the central nervous system depressant effect of alcohol. Participants will, therefore, be advised to be cautious in their intake of alcohol while taking mirtazapine.

Other concomitant care (including switching, discontinuing, or changing the dose of SSRI/SNRI medication, and receipt of psychological therapies) will not be prohibited.

### Adverse Events (AEs)

All AEs (untoward medical occurrences affecting trial participants) will be recorded in the CRF for the duration of the participant’s direct involvement in the trial (12 months). For all events recorded, Centre PIs will record their opinion concerning the nature and severity of the AE, and its relationship to trial therapy.

All SAEs must be reported to UHB (which monitor SAEs on behalf of the sponsor) and the Centre PI by the research team within 24 hours of their knowledge of the event. The chief investigator (CI) and trial manager will also be informed. All SAEs that have not resolved by the end of the trial (that is, by the end of the primary care notes review follow-up period), or that have not resolved upon discontinuation of the participant’s participation in the trial, must be followed until: the event resolves/stabilises/returns to baseline/can be attributed to other factors unrelated to the trial; or it becomes unlikely that additional information can be obtained.

All relevant information about a Suspected Unexpected Adverse Reaction (SUSAR) that occurs during the course of the trial will be reported to the MHRA and the relevant ethics committee by UHB, on behalf of the sponsor as soon as possible (fatal or life-threatening SUSARs will be reported within 7 days, and those which are not fatal or life-threatening within 15 days).

The expectedness of an AE will be determined by whether or not it is listed in the Summary of Product Characteristics, the BNF and study protocol.

### Trial stopping rules

The trial may be prematurely discontinued by the sponsor, CI, regulatory authority or funder on the basis of new safety information or for other reasons given by the Data Monitoring Committee (DMC)/Trial Steering Committee (TSC) regulatory authority or ethics committee concerned.

The trial may also be prematurely discontinued due to lack of recruitment or upon advice from the TSC, which will advise on whether to continue or discontinue the trial and make a recommendation to the sponsor. If the trial is prematurely discontinued, active participants will be informed and no further participant data will be collected.

### Statistical analysis

Analysis and reporting will be in line with Consolidated Standards of Reporting Trials (CONSORT) guidelines [34], with the primary analyses being conducted on an intention-to-treat (ITT) basis. Descriptive statistics will be used to ascertain any marked imbalances in demographic or clinical variables at baseline.

The primary analysis will be the BDI-II score at 12 weeks post randomisation, measured as a continuous variable. The primary analysis will use linear regression to compare the groups as randomised, adjusting for stratification and minimisation variables and baseline measurements of the outcome. Secondary analyses of this outcome will include the BDI-II score at 12 weeks post randomisation as a binary variable representing response, defined as a reduction in depressive symptoms of at least 50 % compared to baseline, and remission, defined as a BDI-II score of less than 10. Secondary analyses will also include additional adjustment for any prognostic variables demonstrating marked imbalance at baseline.

We will conduct pre-planned subgroup analyses to investigate any differential effects according to a number of factors. These will be done by introducing appropriate interaction terms in the regression models. We will carry out these analyses by baseline depression severity (BDI-II) and a five-level measure of the degree of treatment resistance based on duration of symptoms and prior treatment with antidepressants.

In all analyses we will present regression coefficients (or odds ratios for binary outcomes), with 95 % confidence intervals and *p* values.

We will also use repeated measures analyses incorporating the outcomes at 12 and 24 weeks and 12 months post randomisation to examine whether any treatment effects are sustained, diminished or emerge later. This will be investigated formally by the introduction of an interaction between treatment group and time. Finally, we will also investigate the influence of missing data using sensitivity analyses that make different assumptions, such as ‘best’ and ‘worst’ case scenarios, as well as using models to impute missing data [35, 36].

We propose to carry out per protocol analyses at 12 weeks and 12 months comparing individuals who have remained on the trial medication at that follow-up point. Since these analyses are likely to be biased, we will also use the Complier Average Causal Effect (CACE) [37] approach. This provides an unbiased estimate of the treatment effect for those who have complied with the active treatment. This approach would be justified if the characteristics of those who adhered to the placebo differed from those who adhered to mirtazapine. This is plausible as we would expect intolerance of the side effects to be more important for the mirtazapine group and non-response to be more of an issue for the placebo group. If there is differential adherence in the two arms we will also investigate structural mean approaches to take account of this [38] though extensions of CACE to take account of adherence to placebo have also been developed [39].

At 12 and 24 weeks and 12 months, the ITT analysis will compare the randomised groups. By these stages, we would still expect many of those who had responded to mirtazapine to remain on the combination treatment. The ITT analysis will, therefore, provide an estimate of any longer-term benefit attributed to the early response to mirtazapine with an SSRI/SNRI. The interpretation of this will depend upon whether other potentially active interventions are balanced between the groups. We do not expect to see many marked imbalances in other treatments, as our previous trials (IPCRESS [40], CoBalT [18]) have not found this to be a problem. If we do find that the groups differ markedly in the two arms we will investigate any possible impact of this by adjustment for the other interventions in the regression model.

A further sensitivity analysis using CACE methods will be used at 24 weeks and 12 months. If we define ‘compliers’ as those who had continued taking their trial medication up until 12 weeks, we could then estimate the effect of completing a 12 week course of mirtazapine on depression outcomes at the later follow-up points (24 weeks and 12 months).

### Justification of sample size

The primary outcome is BDI-II score as a continuous variable. It is difficult to estimate a clinically important difference in BDI-II score, although the NICE guideline panel for the first depression guideline [4] suggested that this corresponds to about 3 points (0.35 standard deviations) on the Hamilton Depression Rating Scale (HDRS) [41] for non-treatment-resistant patients, and 2 points for those who are treatment-resistant. The equivalent difference to 3 HDRS points on the BDI-II total score would be 3—4 points (standard deviation 10—12 in the CoBalT trial). With 200 participants in each group, we would have 91 % power to detect a difference of 0.33 standard deviations at the 5 % level. Allowing for 15 % loss to follow-up at 12 weeks, we will need to recruit 472 patients.

For our secondary outcome, response rate, defined as a 50 % reduction in symptoms using the BDI-II score, 200 patients in each group would yield 90 % power to detect a difference between 30 and 46 % response, or an odds ratio of 2, at a two-sided 5 % significance level.

We therefore aim to recruit 120 patients from 24 general practices at each of the four recruiting centres (Bristol, Exeter, Keele and Hull).

### Blinding and other forms of bias

Participants, GPs and investigators will be blinded to treatment. The effectiveness of blinding will be assessed by a brief questionnaire asking participants to which arm they believed they had been allocated at the 12-week follow-up.

### Economic evaluation

The economic evaluation will assess the efficiency of mirtazapine plus SSRI or SNRI compared with SSRI or SNRI alone, for primary care patients with TRD. Resource use data will be collected from general practice records review (e.g. number of consultations, prescribed medications and referrals) and a resource use questionnaire (e.g. other community based care, outpatient and inpatient care), completed by participants at 12 and 24 weeks and 12 months. The questionnaire also collects information on employment, time off work, disability payments, informal help, and patient expenditure on healthcare. Publicly available national unit costs will be used to value prescribed medications [17], primary and community care consultations, social services [42] and secondary care [43]. Patients’ time off work and informal help from family and friends will be valued using the human capital approach; we will explore the robustness of our conclusions to other methods for valuing lost productivity [44].

In the primary economic analysis we will estimate the incremental net monetary benefit (iNMB) [45] of combined therapy with mirtazapine at 12 weeks from the perspective of the NHS and social services.

The 12-week time point is selected as clinicians and patients will be blind to treatment allocation. The iNMB estimates whether any additional costs of mirtazapine are justified by improved outcomes (Quality-adjusted Life Years (QALYs)) for patients at conventional thresholds used by NICE (i.e. £20,000 and £30,000 per QALY) [46]. QALYs will be estimated from responses to the EQ-5D-5L controlling for baseline responses [47]. In secondary analyses we will report the iNMB over the 52-week follow-up period and the incremental cost per responder, based on BDI-II, at 12 and 52 weeks. For each analysis, stochastic uncertainty in results will be estimated using confidence intervals and cost-effectiveness acceptability curves. Other uncertainty will be addressed in deterministic sensitivity analyses; specifically, we will explore whether our conclusions are sensitive to the inclusion of patient and wider societal (i.e. lost productivity) costs.

### Qualitative study

A nested qualitative study will: (1) explore patients’ views and experiences of taking either two antidepressant medications or an antidepressant and a placebo; (2) identify patients’ reasons for completing or not completing the study, including withdrawal from study medication; and (3) explore the views of GPs on prescribing a second antidepressant in this patient group.

At the baseline assessment for the main study, individuals will be informed about the qualitative study and be asked to consent to the possibility of being contacted by the qualitative research team to take part in an interview. A purposeful sampling strategy will be used to identify potential interviewees to ensure interviews are held with participants in both arms of the trial, and with individuals in both arms who vary in their levels of adherence. Maximum variation sampling techniques will be used so that patients of different socio-economic background, gender and age are invited for interview. Patients will be sampled across the four centres.

A purposive sample of trial participants will be invited to participate in a semi-structured interview, and perspectives on taking two tablets for depression will be explored. Patients who took the trial medication for at least 12 weeks, and those who stopped, will be sampled. Interviews will be conducted either face to face or by telephone. The interviews will be taped with consent, transcribed, and the transcripts will form the data for analysis. It is anticipated that at least 24 interviews will be needed to achieve category saturation. Interviews will be held with patients after the primary outcome measure has been obtained (at 12 weeks post randomisation) to avoid the possibility of bias that might be introduced by the qualitative interview having a supportive role. Individuals will be interviewed within 8 weeks of their primary outcome measures being taken.

In addition, patients who decline to participate in the trial will be asked if they would be willing to be contacted by a researcher to discuss their reasons for not taking part. Using short semi-structured telephone interviews, their views on the trial, and perspectives on taking two tablets for depression will be explored. The interviews will last 10—30 minutes and will be taped with consent, transcribed, and transcripts analysed. It is anticipated that at least 15 interviews will be needed.

Finally, a purposive sample of GPs (sampled on the basis of practice demographics and size, experience and status (partner, salaried, locum) participating in the trial will be invited to participate in a semi-structured interview which will be taped (with consent). The interviews will be conducted either face to face or by telephone. Interviews will explore perspectives and views of GPs about managing people with depression, use of antidepressants and talking treatments, alternative approaches, switching antidepressants and referral options. The role of national guidelines (particularly about prescribing) in guiding individual management of a patient with depression will be explored. The interviews with be transcribed and the transcripts forming the data for analysis. It is anticipated that between 16 and 20 interviews will be needed to achieve category saturation of the data.

Trial participants and GPs will be interviewed at a time and place that is convenient for them (such as their home, GP surgery or by telephone). Written consent to take part in an interview will be obtained from participants and GPs at the time of face-to-face interviews, or prior to telephone interviews. These interviews will last about an hour. With participant consent, they will be audio-recorded and transcribed verbatim.

Data will be coded by two researchers independently. Thematic analysis will identify and categorise relevant and recurrent concepts within the data set, guided by the research questions of the study. Thematic analysis is guided by a priori concepts but also allows for qualitative data sets to be interrogated in an inductive manner. Themes are produced which unify the conceptual categories [48]. Both data sets will be interrogated and re-analysed against the Normalisation Process Theory framework [49] in order to consider how prescribing two antidepressants may, or may not, be normalised into clinical practice.

### Quality assurance

The trial sponsor takes primary responsibility for ensuring that the design of the study meets appropriate standards and that arrangements are in place to ensure appropriate conduct and reporting. The trial will be run in accordance with Good Clinical Practice (GCP) and current regulatory guidance.

We will employ standard strategies to ensure the quality of the data; for example, a random sample of 20 % of CRFs will be checked, by the trial research team, against entries within the database. Recruiting sites will be asked to perform a self-audit on all entries and provide a return to the Bristol trial centre (who will report to the trial sponsor). A 10 % sample audit will be conducted by the UHB monitoring team.

### Data handling

The database and randomisation system will be designed so as to protect patient information and maintain anonymity. Data will be securely stored in line with the Data Protection Act 1998. The CI is the custodian of the data. Access to the final data set will be restricted to the MIR study team in the first instance. The team will be open to requests by other investigators to access anonymised data.

### Publication policy

An MIR publication policy will be developed and trial publications will be subjected to an independent quality assurance procedure. Publications will conform to the International Committee of International Journal Editors (ICMJE) guidelines for reporting and authorship [50].

### Ethics and regulatory approvals and reporting

The trial will be conducted in compliance all applicable regulatory requirements.

Substantial protocol amendments will be submitted to the Research Ethics Committee (REC) and MHRA, on the agreement of the sponsor. Protocol changes will be disseminated to recruiting sites and GP collaborators as appropriate.

Progress reports will be submitted to the REC and funder as required and will be made available to the DMC and TSC as appropriate. These groups act independently of the investigators and sponsor; further details of these committees are available on request. Annual safety reports will be sent to the MHRA and the REC. An end of study declaration will be submitted to the REC and MHRA. A final report at conclusion of the trial will be submitted to the NIHR, the sponsor, the REC and the MHRA within 1 year of the end of the trial. A summary of the overall trial results will be made available to those participants who have confirmed that they wish to receive them, and to GPs who have recruited to the study.

### Insurance indemnity

The University of Bristol holds professional negligence insurance to cover the legal liability of the university, for harm to participants arising from the design of the research, where the research protocol was designed by the university. The University of Bristol has arranged public liability insurance to cover the legal liability of the University as Research Sponsor in the eventuality of harm to a research participant arising from overall management of the research by the University of Bristol. The other three recruiting sites (Keele, Exeter, Hull) have their own public liability insurance in place for their individual responsibilities.

## Discussion

At this stage of the trial, we anticipate there may be methodological weaknesses in the design, such as inadvertent unblinding due to the common adverse effects of mirtazapine. However, we will address this by asking participants which treatment arm they thought they were allocated, and including this data in the analysis.

The trial has a number of strengths. MIR is a pragmatic primary care study, which will recruit participants from a wide range of primary care practices across the UK. It will also be the largest UK study of two combined antidepressants. We have also included a longer (12-month) follow-up period to reflect the chronicity of the condition. In addition, the qualitative study will tell us more about the acceptability of the intervention to both doctors and patients.

It is important that large pragmatic trials of pharmacological interventions for depression have a placebo arm, since the mean placebo response in treatment trials of major depression has been found to be close to 30 % [51]. We have also used self-report instruments to assess outcomes in order to eliminate the potential for observer bias. Selection bias will be minimised by recruiting participants from a variety of practices based in rural, urban, affluent and deprived areas across the four centres. Exclusion criteria are minimal. These strengths will make the findings more generalisable.

There is substantial unmet clinical need in this population. The intervention is simple, and is likely to be taken-up in primary care if found to be effective and not associated with an unacceptable level of adverse effects such as weight gain in longer-term use. The evidence from the trial will make a contribution to rational and cost-effective prescribing in this important area of patient need, where it is known that current treatments are only partly effective. We think there may be a real opportunity to substantially improve the treatment of depression by combining antidepressants with complementary modes of action. This strategy is only rarely used in primary care at present.

## Trial status

The study began recruiting participants in August 2013 and will be ongoing until the end of September 2015. At the time of writing (August 2015), 106 GP practices have agreed to collaborate with MIR, and 431 participants have been recruited. It is anticipated that data collection will be completed in October 2016 and results will be available in May 2017.

## Abbreviations

AE: Adverse Event
ASEC: Antidepressant Side Effect Checklist
5HT: 5-hydroxytryptamine
BDI: Beck Depression Inventory
BNF: *British National Formulary*
CACE: Complier Average Causal Effect
CCG: Clinical Commissioning Group
CI: Chief Investigator
CIS-R: Clinical Interview Schedule-Revised version
CRF: Case Report Form
DMC: Data Monitoring Committee
GAD-7: Generalised Anxiety Disorder-7 questionnaire
GCP: Good Clinical Practice
GMP: Good Manufacturing Practice
GP: General Practitioner
HDRS: Hamilton Depression Rating Scale
IAPT: Increasing Access to Psychological Therapy
ICD-10: *International Statistical Classification of Diseases and Related Health Problems 10th Revision*
ICMJE: International Committee of Medical Journal Editors
iNMB: incremental net monetary benefit
ITT: intention-to-treat
MHRA: Medicines and Healthcare products Regulatory Authority
MHRN: Mental Health Research Network
MAO: monoamine oxidase
NA: noradrenaline
NICE: National Institute for Health and Care Excellence
NIHR: National Institute for Health Research
PHQ: Patient Health Questionnaire
QALY: Quality-adjusted Life Year
QP: Qualified Person
REC: Research Ethics Committee
SAE: Serious Adverse Event
SSRI: selective serotonin reuptake inhibitor
SNRI: serotonin and noradrenaline reuptake inhibitor
SUSAR: Suspected Unexpected Serious Adverse Reaction
TRD: treatment-resistant depression
TSC: Trial Steering Committee
UHB: University Hospitals Bristol
